# Supplementary material for: Hybrid Models and Biological Model Reduction with PyDSTool
Source: PLoS Comput Biol. 2012 Aug 9;8(8):e1002628. doi: 10.1371/journal.pcbi.1002628 (PMC3415397; doi:10.1371/journal.pcbi.1002628)
Supplement: Text S4 — Complete source code for the PyDSTool package (version 0.88.120504). Includes API documentation and help files linking to web pages. This file is identical to the current public release on Sourceforge.net. (ZIP) [file pcbi.1002628.s004.zip › PyDSTool/html/PyDSTool.ModelConstructor'.EvMapping-class.html]

xml version="1.0" encoding="ascii"?


PyDSTool.ModelConstructor'.EvMapping


| Home | Trees | Indices | Help | PyDSTool |
| --- | --- | --- | --- | --- |

Package PyDSTool ::
Module ModelConstructor' ::
Class EvMapping

[hide private]

[frames] | no frames]

# Class EvMapping

source code

```
object --+
         |
        EvMapping
```

---

```
Event mapping class, for use by makeModelInfoEntry and, when
instantiated, the Model class.

assignDict maps its values onto the variable or parameter named by the
key. To use the simple syntax in these assignments, either the 'model'
argument or the 'infodict' argument must be provided, the first taking
preference if both are provided. An instantiated Model object must be
provided with the 'model' argument in order to name these variables and
parameters without further qualification. A dictionary with keys 'vars'
and 'pars' must provide lists of variable and parameter names for the
'infodict' argument. Use this argument with ModelConstructor when an
instantiated model is not available. With either of these arguments,
assignments must be given in the (key, value) form: "a", "1 + a*k/2"

Without the model argument, assignments must be given in the (key, value) form:
 "xdict['a']", "1+xdict['a']*pdict['k']/2"

defStrings (list of valid python statements) overrides assignDict if supplied at
initialization, to permit full flexibility in the contents of the
event mapping function. These strings must use "xdict" and "pdict" to reference
the variables and parameters, respectively.

Use activeDict to map named events to a given setting for 'active' (Boolean).
```


|  |  |
| --- | --- |
| Instance Methods | [hide private] |

|  |  |
| --- | --- |
| \_\_cmp\_\_(self, other) | source code |

|  |  |
| --- | --- |
| \_\_getstate\_\_(self) | source code |

|  |  |
| --- | --- |
| \_\_init\_\_(self, assignDict=None, defString=`'``'`, activeDict=None, model=None, infodict=None)  x.\_\_init\_\_(...) initializes x; see x.\_\_class\_\_.\_\_doc\_\_ for signature | source code |

|  |  |
| --- | --- |
| \_\_setstate\_\_(self, state) | source code |

|  |  |
| --- | --- |
| makeCallFn(self) | source code |

**Inherited from `object`**:
`__delattr__`,
`__getattribute__`,
`__hash__`,
`__new__`,
`__reduce__`,
`__reduce_ex__`,
`__repr__`,
`__setattr__`,
`__str__`


|  |  |
| --- | --- |
| Properties | [hide private] |

**Inherited from `object`**:
`__class__`


|  |  |
| --- | --- |
| Method Details | [hide private] |

|  |  |
| --- | --- |
| \_\_init\_\_(self, assignDict=None, defString=`'``'`, activeDict=None, model=None, infodict=None)  *(Constructor)* | source code |

x.\_\_init\_\_(...) initializes x; see x.\_\_class\_\_.\_\_doc\_\_ for
signature

Overrides: object.\_\_init\_\_: *(inherited documentation)*

  


| Home | Trees | Indices | Help | PyDSTool |
| --- | --- | --- | --- | --- |

|  |  |
| --- | --- |
| Generated by Epydoc 3.0.1 on Fri May 4 15:24:07 2012 | http://epydoc.sourceforge.net |
